# Postural threat during walking: effects on energy cost and accompanying gait changes

**DOI:** 10.1186/1743-0003-11-71

**Published:** 2014-04-22

**Authors:** Trienke IJmker, Claudine J Lamoth, Han Houdijk, Lucas HV van der Woude, Peter J Beek

**Affiliations:** 1 MOVE Research Institute Amsterdam, Faculty of Human Movement Sciences, VU University Amsterdam, van der Boechorststraat 9, 1081 BT Amsterdam, the Netherlands; 2 Heliomare Research & Development, Relweg 51, 1949 EC Wijk aan Zee, the Netherlands; 3 University of Groningen, University Medical Center Groningen, Center for Human Movement Sciences, Center for Rehabilitation, Antonius Deusinglaan 1, 9713AV Groningen, the Netherlands; 4 Brunel University, School of Sport and Education, Kingston Lane, Middlesex UB8 3PH, Uxbridge, UK

## Abstract

**Background:**

Balance control during walking has been shown to involve a metabolic cost in healthy subjects, but it is unclear how this cost changes as a function of postural threat. The aim of the present study was to determine the influence of postural threat on the energy cost of walking, as well as on concomitant changes in spatiotemporal gait parameters, muscle activity and perturbation responses. In addition, we examined if and how these effects are dependent on walking speed.

**Methods:**

Healthy subjects walked on a treadmill under four conditions of varying postural threat. Each condition was performed at 7 walking speeds ranging from 60-140% of preferred speed. Postural threat was induced by applying unexpected sideward pulls to the pelvis and varied experimentally by manipulating the width of the path subjects had to walk on.

**Results:**

Results showed that the energy cost of walking increased by 6-13% in the two conditions with the largest postural threat. This increase in metabolic demand was accompanied by adaptations in spatiotemporal gait parameters and increases in muscle activity, which likely served to arm the participants against a potential loss of balance in the face of the postural threat. Perturbation responses exhibited a slower rate of recovery in high threat conditions, probably reflecting a change in strategy to cope with the imposed constraints. The observed changes occurred independent of changes in walking speed, suggesting that walking speed is not a major determinant influencing gait stability in healthy young adults.

**Conclusions:**

The current study shows that in healthy adults, increasing postural threat leads to a decrease in gait economy, independent of walking speed. This could be an important factor in the elevated energy costs of pathological gait.

## Background

Walking requires metabolic energy, primarily to generate muscle force and work for body weight support, propulsion and leg swing [1-3]. Balance control is another important factor that adds to the energy requirements of walking, but has been studied less extensively. Recent studies have shown that external stabilization or facilitation of balance control while walking can cause a reduction in the energy cost of walking up to 7% in healthy subjects [4-7] and even more so in stroke patients [8]. This indicates that active balance control during walking inflicts a metabolic load, which may be larger for patient populations than healthy subjects.

While these stabilization studies provide valuable insight into the energy cost of balance control during walking, the methodology used is confined to the role of balance during walking in a perturbation free environment. During everyday walking balance control is often threatened by the environment due to external perturbations, such as a push or pull, or challenges with respect to the walking surface (such as walking on a narrow ridge). Anticipating such conditions of postural threat has been shown to induce changes in the gait pattern in terms of kinematics and EMG activity. Some general changes in the face of threatening conditions are a reduced stride time and stride length and an increased step width [9-11], as well as increased muscle activity and muscle co-activation [12,13]. Thus far, effects of postural threat on the response to perturbations during gait have not been investigated extensively and might vary depending on perturbation type, magnitude, timing and direction [14,15]. In all likelihood, the responses to postural threat, which may serve to reduce the risk of falling and enhance safety, require additional muscular effort and thus carry an additional metabolic load. The magnitude of this metabolic demand however, is unknown.

To date, two studies have been published that experimentally manipulated the perceived level of postural threat and evaluated the effect on energy cost during locomotion. One study manipulated feelings of postural threat during running by elevating a treadmill above the ground, and found that this increased the energy cost of running by approximately 3% [16]. Similarly, in a study on downhill treadmill walking under the threat of perturbations a significant increase in energy cost was found [17]. While these studies demonstrate that postural threat could affect metabolic cost, they do not necessarily apply to level walking. Therefore, in the current study we examined the potential effect of postural threat on the energy cost and the associated gait changes for level walking with actual perturbations.

In addition, since walking speed is known to be a major factor in the organization of human walking and the adaptation of walking to environmental contingencies including postural threat, we were interested in determining if and how the metabolic cost of balance control during conditions of postural threat is influenced by walking speed. Changes in walking speed in reaction to postural threat are equivocal, with some studies showing a decreased speed in the face of postural threat [18] and others showing no change or even increases in speed [9,19].

The aim of the present study was thus to examine how increasing levels of postural threat, induced by a combination of discrete mechanical perturbations and path width constraints, affect the energy cost of level walking, as well as the accompanying gait parameters, muscle activity and perturbation responses. In addition, we examined if and how these effects are dependent on walking speed.

## Methods

### Study population

Fifteen healthy young adults participated in the experiment (7 male, 8 female; age 26.6 ± 5.0 yrs; body height 1.77 ± 0.1 m; body mass 68.1 ± 10.5 kg; mean ± SD). Exclusion criteria comprised balance impairments and cardiovascular, neurologic or orthopedic limitations that could interfere with the study protocol. All subjects signed a written informed consent prior to participation and the study protocol was approved by the local Ethical Committee of the Faculty of Human Movement Sciences at the VU University Amsterdam.

### Study design

Subjects completed 28 five minute walking trials on a treadmill, consisting of 4 postural threat conditions performed at 7 different walking speeds. Trials were performed over two separate days and with a minimum of 2 minutes rest between trials to avoid exertion. A habituation trial of 5 minutes walking at preferred speed was performed on both days prior to the first walking trial. For each of the 4 postural threat conditions at a given speed 2 conditions were performed on day one and the other 2 on day two, such that both days were approximately equal in intensity. The 14 experimental trials per day were executed in a random order.

The postural threat conditions were created through a combination of the presence of external mechanical perturbations and path width constraints. The variations in path width were imposed in order to exacerbate the postural threat induced by the mechanical perturbations in a graded fashion. In particular, the following four postural threat conditions were applied: 1) No Threat (NT); unperturbed walking on a wide path (1.0 m); 2) Low Threat (LT); walking with perturbations on a wide path; 3) Medium Threat (MT); walking with perturbations on a path of intermediate width (0.5 m); and 4) High Threat (HT); walking with perturbations on a small path (0.3 m). Subjects were instructed to walk within the imposed path at all times.

**Figure 1:**
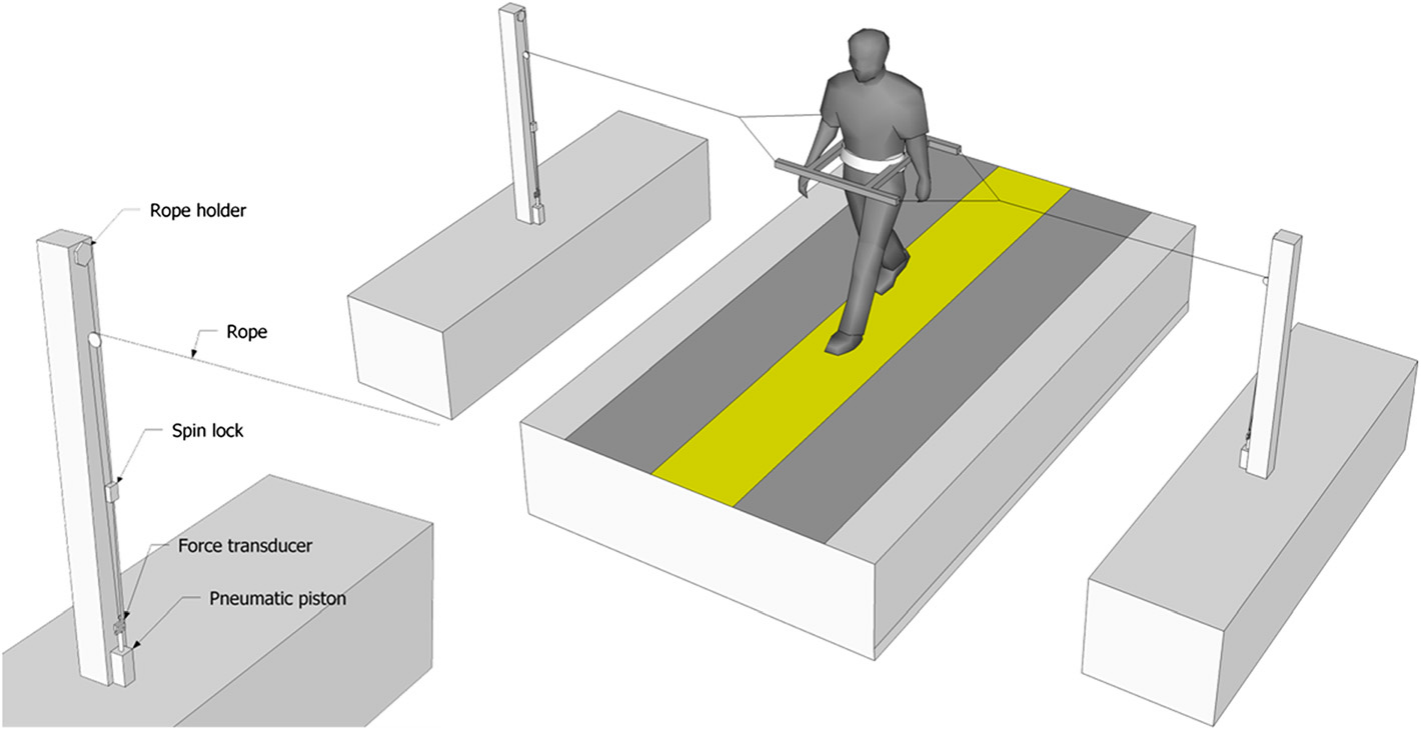
Schematic representation of experimental set-up.

Mechanical perturbations consisted of an unexpected sideward pull to the pelvis in the direction of the swing leg applied approximately at midswing. The perturbation was controlled by a computer and applied via a rope connected to a frame worn around the pelvis, which allowed normal arm swing. On a predefined kinematic cue corresponding to midswing as detected by a custom written computer program, the normally free running rope was blocked by a pneumatic latch, and a pneumatic piston at the other end of the rope went down, causing a shortening of the rope of ~0.1 m, corresponding to a sideward pull to the pelvis (Figure 1). A force transducer attached to the piston and rope of the perturbation device measured the resulting perturbation force, which is dependent both on the forces applied by the pistons (the actual perturbation force) as well as the compliance of the subjects (e.g. the tension in the ropes). Twelve perturbations were applied at random instants with 3 perturbations during the final 2 minutes of each trial, during which energy cost was determined, so as to limit the effect of perturbation responses on energy cost.

Path width was enforced by instructing subjects to stay within a yellow rectangular path, which was projected onto the treadmill belt by a beamer placed in front of the treadmill. Path widths were chosen such that in the MT condition (0.5 m) subjects would be able to use a mediolateral stepping strategy in response to perturbations although they needed to be careful not to step too far to the side; in the HT condition (0.3 m) they could walk with a normal step width but no longer make use of a stepping strategy in the face of perturbations. Subjects were made aware of the postural threat condition prior to each trial.

Trials were executed at 60-70-80-100-120-130-140% of the preferred speed of the subject. Preferred speed was determined prior to the first experimental trial following a previously described method [20]. Subjects wore a safety harness during all trials.

### Data collection and analysis

#### Energy cost

Energy cost was calculated from oxygen uptake (${\dot{V}O}_{2}$, ml · min^−1^) and respiratory exchange ratio (RER) obtained from breath-by-breath gas analysis (Cosmed K4*b*^2^, Italy). To ensure steady state oxygen consumption rate, only the final two minutes of each trial were used for the analysis. Gross energy expenditure (E_gross_) was calculated using the following formula [21]:

(1) $$E_{\text{gross}}\left(J\cdot {\text{min}}^{-1}\right)=\left(4.960\;\cdot \;\text{RER}+16.040\right)\;\cdot \;{\overset{\cdot }{V}O}_{2}$$

Net energy expenditure E_net_ was calculated by subtracting resting metabolism in supine position from the gross metabolic energy expenditure during walking. Net energy cost (EC, J · kg^−1^ · m^−1^) was obtained by dividing E_net_ by body mass (kg) and walking speed (m · min^−1^).

#### Gait parameters

Stride time, stride length, step width and their within-trial variability were calculated from kinematic data from the heel markers, recorded with an Optotrak motion analysis system (Northern Digital Inc., Canada) sampled at 100 Hz. Data were filtered with a bi-directional low-pass Butterworth filter with a cut-off frequency of 10 Hz. Instants of foot contact were determined from local minima in the vertical heel marker data.

Stride time was defined as the time difference (ST) between two consecutive homolateral foot contacts. Stride length (SL) was calculated by adding left and right consecutive step lengths, defined as the anterior-posterior distance between the two heel markers at foot contact. Step width (SW) was defined as the absolute mediolateral distance between the heel markers at two consecutive foot contacts. The standard deviation of stride times (sdST) stride lengths (sdSL) and step widths (sdSW) within each trial were used to quantify the variability of these parameters. Gait parameters were calculated over the entire trial, but the first two complete strides following a perturbation were removed from this analysis to correct for recovery strides.

#### Perturbation responses

Responses to the perturbations were quantified by analyzing the deviation from and rate of return towards the nominal gait cycle using 3D linear accelerations (*g*) and 3D angular velocities (deg · sec^−1^) measured with a tri-axial accelerometer and gyroscope (Dynaport Hybrid, McRoberts, the Netherlands), which was attached to the trunk at the level of L5 [22]. Only perturbations with >10 perturbation-free strides prior to and >5 perturbation-free strides after the perturbation were used for the analysis, resulting in an average of 6 valid perturbations per trial.

Data were filtered with a second order Butterworth filter with a cut-off frequency of 15 Hz. Instants of foot contact were determined from anterior-posterior (AP) trunk accelerations. Instants of perturbations were estimated by detecting peaks in the mediolateral (ML) acceleration data. Data were time normalized so that a single stride consisted of 100 samples. A nominal cycle of the strides prior to the perturbation was computed by averaging the last 10 strides before the perturbation, and standard deviations across strides were calculated for each percentage of this nominal cycle.

Thereafter, for each of the six dimensions (*d*; 3D linear accelerations and 3D angular velocities), the distance between the actual signal (*x*) and the nominal signal ($\bar{x}$) was computed from the last foot contact prior to the perturbation (t = 0) until the 5^th^ foot contact after the perturbation (t = 500). Distances were normalized to the standard deviation (*SD*) of the nominal signal at that point in the stride. A resultant normalized distance (*D*) was calculated by taking the square root of the sum of squares of the normalized distances of all 6 dimensions:

(2) $$D\left(t\right)=\sqrt{\sum_{d}^{6}{\left(\left(x_{\left(t\right)}\;-\;{\bar{x}}_{\left(i,t\right)}\right)/SD_{\left(i,t\right)}\right)}^{2}},$$

where *t* is the normalized time starting from the last foot contact prior to the perturbation and *i* is the % of stride time at point *t*.

**Figure 2:**
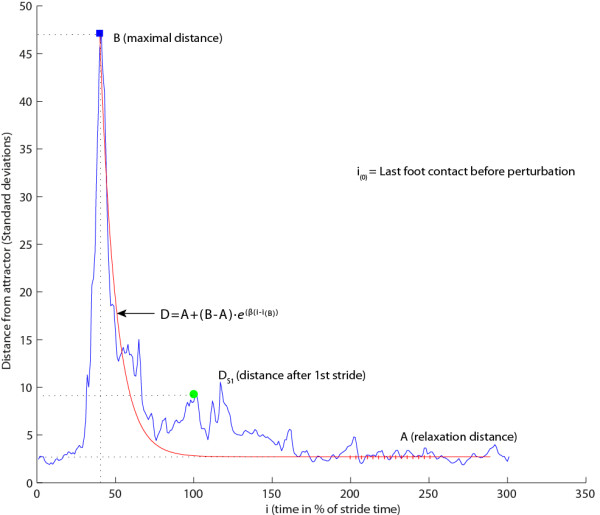
**Explanation of perturbation responses.** B = maximal deviation; β = rate of return (based on fit); D_S1_ = distance after 1 stride.

An example of *D*, as a function of time, can be seen in Figure 2. From *D (t)*, three outcome parameters were calculated to quantify the perturbation responses: 1) *B* is the maximal deviation indicating the disruption of the gait pattern caused by the perturbation; 2) *D*_*S1*_ is the normalized distance at the next homolateral foot contact, quantifying the recovery after one stride; and 3) *β* is the exponential decay quantifying the rate of return towards the nominal cycle based on fitting an exponential function to the data (formula 3):

(3) $$D_{\text{fit}}\left(t\right)=A+\left(B\;-\;A\right)\;\cdot \;e^{-\beta \left(t-t_{B}\right)}$$

where *A* is the relaxation distance quantified as the average deviation from 200–250 time points after *B*. Large values for *β* correspond to a faster return towards the limit cycle.

#### Muscle activity

Muscle activity was recorded using surface electromyography (TMSI, the Netherlands) from *m*. rectus femoris (RF), *m*. biceps femoris (BF), *m*. tibialis anterior (TA), *m*. gastrocnemius medialis (GM) and *m*. peroneus longus (PL) of the right leg. Data were high pass filtered (20 Hz), rectified, and low pass filtered (10 Hz) to create a linear envelope. For each subject the EMG signals were normalized to the mean amplitude for each muscle during the habituation trial of the same day [23]. Average normalized EMG amplitudes and co-contraction indices (CCI) were calculated over 20 consecutive perturbation-free strides during the final 2 minutes of each walking trial. Co-contraction index (in %) was calculated as the common area of activity of two antagonistic muscles for GM_TA, TA_PL, and RF_BF [24] (eq. 4).

(4) $$\mathrm{CCI}\left(\%\right)=2\;\cdot \;\frac{\int \min\left(m1,m2\right)}{\int \left(m1+m2\right)}\;\cdot \;100\%$$

where *m1* and *m2* are the full wave rectified EMG profiles of the antagonistic muscle pair averaged over 20 consecutive strides and min (*m1,m2*) is the minimum of the two profiles.

### Statistical analysis

A two-way repeated analysis of variance with Postural threat (4 levels) and Speed (7 levels) as within-subject factors was conducted to evaluate the effect of postural threat, and the interaction of walking speed and postural threat on energy cost, spatiotemporal gait parameters, muscle activation and perturbation responses. Since we were interested in the influence of speed on the effect of postural threat on the outcome variables, but not in the effect of speed per se, main effects of speed will not be reported for the sake of legibility. In the case of violation of the assumption of sphericity the Greenhouse-Geisser correction was applied. Where applicable, a simple contrast was used to further investigate the effect of postural threat using the NT or the LT (for the perturbation responses) condition as reference category. Level of significance for all statistical analyses was set at *p* < 0.05.

## Results

The average preferred walking speed of the subjects was 1.3 m · s^−1^. Accelerometer data for one of the subjects were lost due to malfunctioning of the equipment; therefore this subject was removed from the analysis of the perturbation responses. Peak perturbation force was not influenced by walking speed and was 256 ± 21 N (mean ± standard deviation). None of the subjects fell or made use of the safety harness during the trials.

### Energy cost

**Figure 3:**
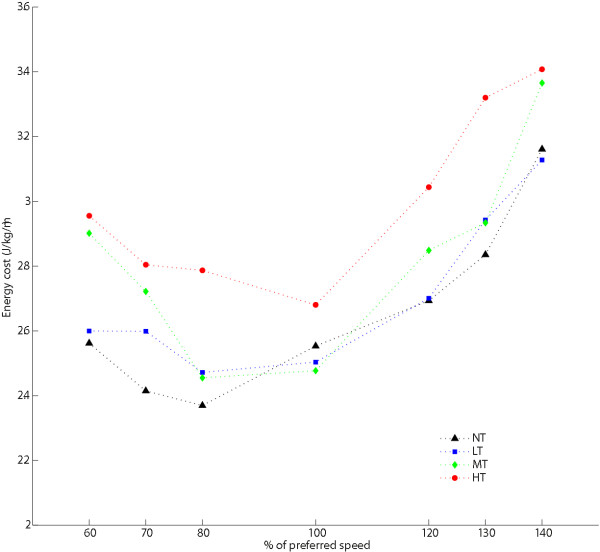
**Energy cost.** (N = 15) Separate lines represent different postural threat conditions: NT (black triangles) = no threat; LT (blue squares) = Low threat, wide path with perturbations; MT (green diamonds) = Medium threat, intermediate path with perturbations; HT (red circles) = High threat, small path with perturbations.

A statistically significant main effect of postural threat on the energy cost of walking was found. Energy cost was on average 6.7% higher in the MT (*p* < .05) and 13.6% higher in the HT condition (*p* < .01) compared to the NT condition (Figure 3). The difference in the LT condition (1.6%) was not significant (*p* = .178).

No significant Speed × Postural threat interaction was found, although the average increases in energy cost tended to be largest in the most extreme speed conditions.

### Gait parameters

**Figure 4:**
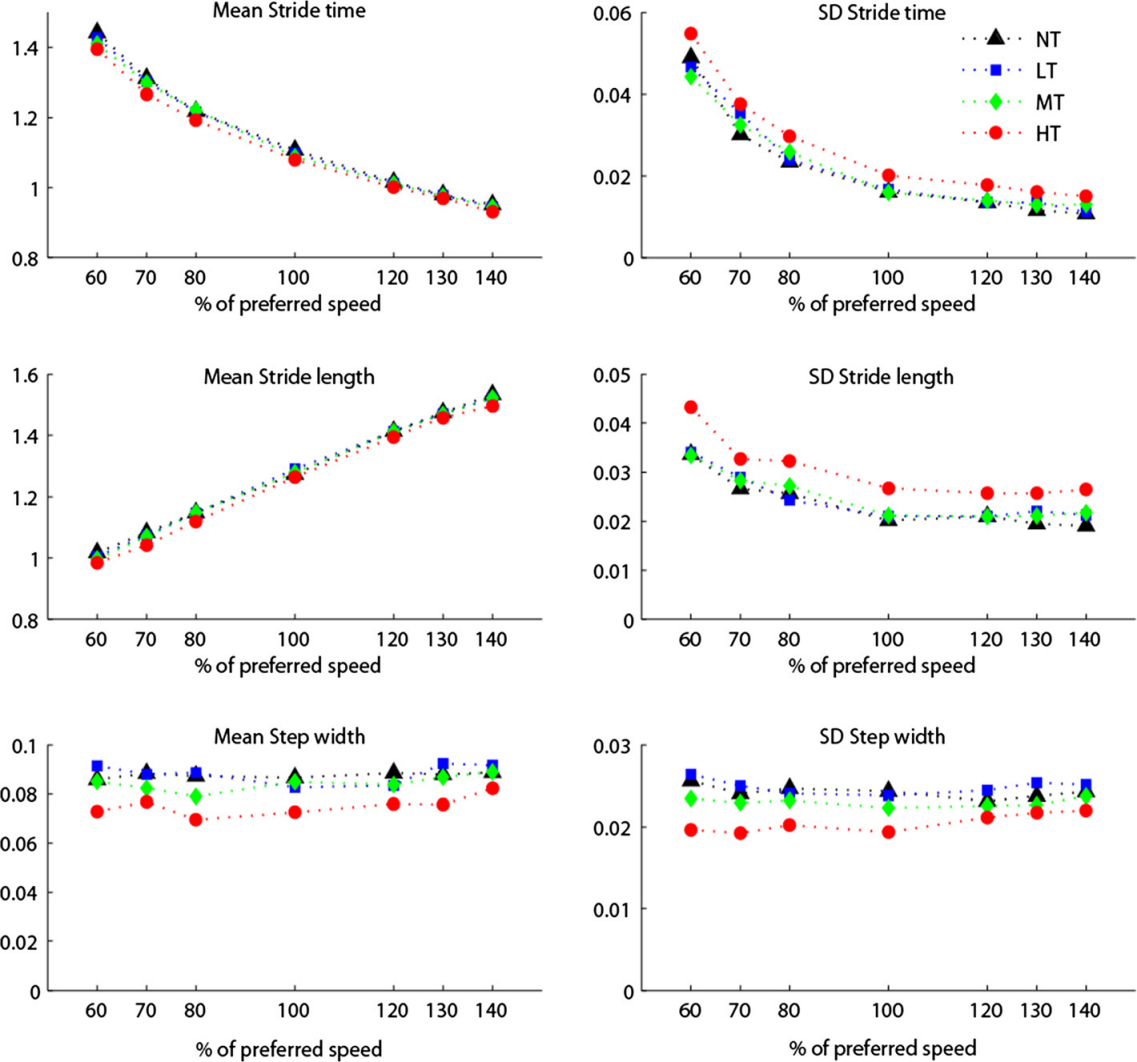
**Spatiotemporal gait parameters.** (N = 15) Separate lines represent different postural threat conditions: NT (black) = no threat; LT (blue) = Low threat, wide path with perturbations; MT (green) = Medium threat, intermediate path with perturbations; HT (red) = High threat, small path with perturbations.

Postural threat caused statistically significant changes in all gait parameters (Figure 4). Planned contrasts showed that in the HT condition subjects walked with slightly faster (2.6%), shorter (2.2%), and narrower steps (13%) compared to the control condition (*p* < .01 for all parameters). The decrease in ST was also significant for the MT condition (1.2%, *p* < .01). Furthermore, for the HT condition, sdST and sdSL increased (25% and 30% respectively, *p* < .01), whereas sdSW decreased (15% *p* < .05).

No significant Speed × Postural threat interactions were found.

### Perturbation responses

**Figure 5:**
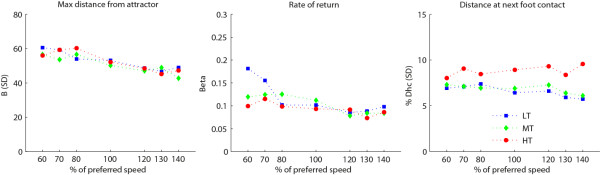
**Perturbation responses.** (N = 14) Separate lines represent different postural threat conditions: NT (black) = no threat; LT (blue) = Low threat, wide path with perturbations; MT (green) = Medium threat, intermediate path with perturbations; HT (red) = High threat, small path with perturbations.

Increasing postural threat showed a statistically significant main effect on *β* and *D*_*S1*_: for walking in the HT condition recovery was slower compared to the LT condition as indicated by a lower rate of return (*β; p* < .05), and a larger distance at the next stride (*D*_*S1*_; *p* < .01) (Figure 5).

No significant Speed × Postural threat interactions were found.

### Muscle activity

**Figure 6:**
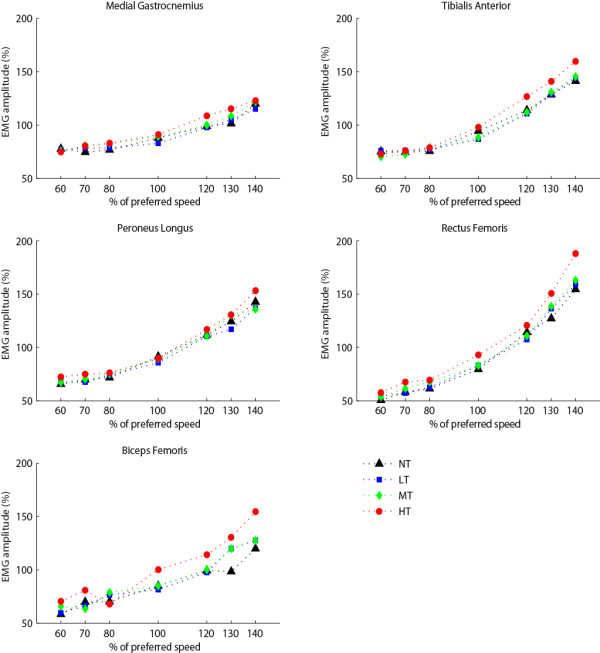
**EMG amplitude.** (N = 15) Separate lines represent different postural threat conditions: NT (black) = no threat; LT (blue) = Low threat, wide path with perturbations; MT (green) = Medium threat, intermediate path with perturbations; HT (red) = High threat, small path with perturbations.

**Figure 7:**
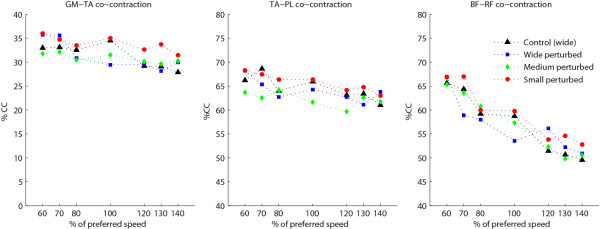
**Co-contraction indices.** (N = 15) Separate lines represent different postural threat conditions: NT (black) = no threat; LT (blue) = Low threat, wide path with perturbations; MT (green) = Medium threat, intermediate path with perturbations; HT (red) = High threat, small path with perturbations.

Statistically significant increases in muscle activity and CCI in response to postural threat were found. Planned contrasts showed muscle activity of GM and BF increased by 4.7% and 9.6% respectively for the MT condition compared to the NT condition (*p* < .05). For the HT condition muscle activity of GM (6.7%), TA (6.7%), BF (27%), and RF (21.8%) were increased compared to the NT condition (*p* < .05 for all muscles, Figure 6). Also, GM_TA and RF_BF co-contraction indices were increased for the HT condition compared to the NT condition (11.7% and 6.9% respectively *p* < .05, Figure 7).

No significant Speed × Postural threat interactions were found.

## Discussion

This study aimed to determine the influence of postural threat on the energy cost of walking, as well as associated changes in gait parameters, muscle activity and perturbation responses. In addition, we sought to determine if and how these effects are dependent on walking speed. In general, the results showed that postural threat significantly increased the energy cost of walking, and resulted in marked effects on gait parameters, muscle activity and recovery responses after perturbations. Walking speed did not influence the energy cost of walking under postural threat, nor had it any effect on the changes in the gait pattern in response to the imposed threat, given the absence of any significant interactions with postural threat.

### The effect of postural threat

The energy cost of walking increased with the level of postural threat, with the largest change (13.6%) found in the high threat condition. Concomitant changes in spatiotemporal gait parameters and muscle activity were evident. In all likelihood, these changes reflect an attempt to arm oneself against the postural threat and are responsible for, or at least contribute, to the elevated energy cost of walking.

In accordance with other studies in which balance control was threatened, subjects consistently took slightly shorter and faster steps in the high threat condition [9]. Although these changes were minimal (on average 2.2% and 2.6% decrease in stride length and stride time), such gait adaptations may help to improve backward and mediolateral margins of stability [25]. At the same time, the variability of stride time and length increased considerably (25% and 30% respectively). Increased movement variability has been interpreted as a sign of instability [26,27], but could also be a means to control stability through variations in timing and position of foot contact [5,28]. Together, the changes in mean and variability of these parameters represent a deviation from the preferred step frequency – step length combination, which has been shown to induce a greater energetic cost [29,30].

In addition to these changes in stride time and length, postural threat elicited a decrease in step width and step width variability (13% and 15%). This decrease in step width seems counterproductive for gait stability since it decreases the mediolateral margins of stability [31]. However, it also ensures a margin between the lateral border of the foot and the lateral border of the projected path, thereby providing room for maneuverability in the event of a perturbation. The decrease in step width and step width variability could also contribute to the elevated energy cost since steps narrower than preferred have been shown to increase the energy cost of walking [3,32]. However, compared to these studies, the magnitude of the changes in step width observed here will only explain a small part of the observed increase in energy cost. Alternatively, the reduced step width and the restriction on the stepping strategy for balance control at the high threat condition requires the selection of other possibly less efficient balance control mechanisms, such as trunk motion, or an ankle or hip strategy [33].

An increase in muscle activity, requiring increased ATP turnover, is likely an important contributor to the elevated energy cost of walking in the highest threat conditions. Although EMG was measured in only a limited set of muscles, muscle activity of the main muscles of the lower leg was indeed increased in the medium and high threat condition compared to normal walking. This increased muscle activation could be regarded as increased phasic activity required for the altered foot placement or alternative control strategies, such as ankle/hip motion. In addition, it might be related to tonic activity to increase joint stiffness, as evidenced by increased co-activation.

Besides the changes in energy cost and in the nominal gait pattern, the high threat condition also induced changes in perturbation responses. Although the maximal deviation from the nominal gait cycle did not differ between postural threat conditions, the recovery time was longer in the high threat condition, as indicated by a lower value for *β* and a larger distance from the attractor after one stride. This probably reflects a change in recovery strategy. The small path in the high threat condition does not only amplify the threat from the perturbations, but also limits the use of a foot placement strategy to control balance. Hence, other less efficient strategies such as hip, trunk or arm countermotion need to be employed. Besides being less effective in terms of the rate of recovery, these strategies might also be less economic, although in our study this effect will have been limited due to the small number of perturbations in the time period over which energy cost was calculated.

In accordance with studies on standing postural control, the observed changes in energy cost, spatiotemporal parameters and muscle activity appear to be proportional to the level of postural threat [34]. Only in conditions where the potential consequences of losing balance were large enough (i.e. the highest threat conditions), adaptations in the gait pattern were made, resulting in less economic walking. In elderly, especially those with fear of falling or impairments in balance control [35,36], normal walking might already pose a postural threat large enough to elicit gait changes similar to those seen in young adults in conditions of postural threat [37-41], resulting in a reduced economy. Therefore, postural threat might explain in part the elevated energy cost of walking in these populations [42,43]. However, the current results cannot be generalized to these populations and further research is necessary to validate these suggestions.

### The influence of walking speed on the effect of postural threat

It is generally believed that reducing walking speed represents a strategy to enhance gait stability and balance control during walking in the face of postural threat. However, while postural threat affected the energy cost, spatiotemporal gait parameters and muscle activity, no significant interactions with walking speed were evident. Together, these results suggest that walking speed is not a major factor influencing gait stability and the effort for balance control in this group of healthy young subjects. This is consistent with other recent studies [9,7,44], and hence contributes to the growing evidence against the hypothesis that slow walking is more stable.

### Limitations

Several limitations of the current study should be considered. Firstly, postural threat can be imposed in many ways, and some of the observed changes in the gait pattern in this study may not generalize to other types of postural threat. However, this does not invalidate the main finding of the study that postural threat can have a substantial effect on the metabolic cost of walking.

Secondly, the current set-up does not permit a conclusion on whether or not the changes in gait parameters and muscle activation had any beneficial contribution to the recovery parameters in terms of the magnitude of deviation or rate of recovery, as this would require a control condition at the small path without such adaptations. Future studies could be directed at extricating whether the changes in the gait pattern induced by the postural threat also lead to altered/beneficial recovery responses. Moreover the current experiment does not allow us to reveal the individual contribution of all of the observed changes in the gait pattern to the increased metabolic cost. Future studies should be designed to unravel this.

## Conclusion

Postural threat during human walking elicits a metabolic cost. If balance control is challenged and consequences of a loss of balance are sufficiently large, the metabolic effort for balance control increases. This increase in energy cost is accompanied by changes in spatiotemporal gait parameters and muscle activity that may be understood as an attempt to enhance gait stability and/or the selection of alternative (but less economic) balance control strategies. However, the contribution of the specific changes in the gait pattern to the increased energy cost cannot be established yet. The absence of a significant interaction of speed and postural threat suggests that walking speed does not have a major influence on the metabolic effort for balance control. The current study clearly shows that postural threat not only alters movement execution in terms of spatiotemporal characteristics, muscle activation and perturbation responses but also leads to a decrease in movement economy. This should be kept in mind as an essential feature that could in part explain the increased energy cost of elderly and pathological gait, where stability might be prioritized over economy.

## Competing interests

The authors declare that they have no competing interests.

## Authors’ contributions

All authors contributed significantly to this work and were involved in the development of the concept and set-up of the experiment. CL and HH supervised the project. TIJ collected the data, performed the analysis and wrote the main paper. All authors discussed and commented on the manuscript at all stages. All authors read and approved the final manuscript.
